# BIOLOGICAL AGING IS ASSOCIATED WITH INCREASED MONOCYTE INFLAMMATORY ACTIVITY IN OLDER ADULTS

**DOI:** 10.1093/geroni/igz038.3315

**Published:** 2019-11-08

**Authors:** Juliette Tavenier, Line J H Rasmussen, Morten B Houlind, Aino L Andersen, Anne Langkilde, Jan Nehlin, Janne Petersen, Ove Andersen

**Affiliations:** Clinical Research Centre, Copenhagen University Hospital, Hvidovre, Hvidovre, Denmark

## Abstract

Chronic inflammation is thought to play a central role in biological aging. However, the causes of chronic inflammation are not fully elucidated. We hypothesized that a dysregulation in monocyte inflammatory activity may contribute to chronic inflammation and biological aging. There are no validated methods for Biological Age (BA) estimation. Therefore, we also hypothesized that older adults with a recent ED (Emergency Department) admission had a higher BA compared to age-matched older adults without a recent ED admission. Two groups of older adults were enrolled: a “high BA”-group who were discharged from the ED four weeks preceding data collection (n=52), and a “low BA”-group consisting of age and sex matched participants without ED admission within the two years preceding data collection (n=52). We assessed NF-κB phosphorylation (Ser529) and NLRP3 inflammasome levels in monocytes using flow cytometry staining of whole blood. Preliminary analyses showed that participants had a median age of 74.8 (IQR: 70.7–82.0) years, 48% were women. Participants in the high-BA group had reduced lower body strength (30 seconds chair stand test p=0.02 and 4 meters gait speed p=0.001) and cognitive function (Digit Symbol Substitution Test p=0.001 and Trail Making Test p=0.002) compared to the low-BA group. Monocytes of participants in the low BA group had lower constitutive p-NF-κB (p< 0.0001) and NLRP3 (p=0.0001) median fluorescence intensity compared to the high BA group. Increased monocyte inflammatory activity assessed by p-NF-κB and NLRP3 was associated with a higher BA. We will investigate associations between monocyte inflammatory activity and markers of chronic inflammation.

